# Possible Influence of Resistance to Malaria in Clinical Presentation of Rheumatoid Arthritis: Biological Significance of Natural Selection

**DOI:** 10.1155/2012/670579

**Published:** 2012-11-14

**Authors:** Fabio Bonilla-Abadía, Gabriel J. Tobón, Carlos A. Cañas

**Affiliations:** ^1^Division of Rheumatology, Department of Internal Medicine, Fundación Valle del Lili and ICESI University School of Medicine, Cali, Colombia; ^2^Laboratory of Immunorheumatology, Carrera 98 N. 18-49, Cali, Colombia

## Abstract

Rheumatoid arthritis (RA) is a common autoimmune disease that affects all ethnic groups. Genetic factors, mainly HLA alleles, are highly associated with increased risk to develop RA. However, there are few available data about the role of these genetic polymorphisms in the prevalence or severity of RA in the Afrodescendant population, who have evolutionarily and by natural selection developed mutations that allowed them to acquire resistance to infectious diseases like malaria. Some of the mechanisms, by which this resistance was developed as a product of natural selection, are involved in different forms of immunological response, many of them of a well-known importance in the pathophysiology of RA. This paper focuses on presenting the known mechanisms of resistance to malaria and their possible contribution to the pathophysiology of RA, including “loss-of-function” mutations, lack of expression of chemokine receptors, decrease of immune complexes clearance by asplenia, or increase of immune reactivity mediated by B cells, among other mechanisms in this special group of patients.

## 1. Introduction

Rheumatoid arthritis (RA) is a common disease that affects all ethnic groups with a prevalence estimated between 0.5 and 1% of the general population [1]. It is a chronic autoimmune disorder with a systemic character and several clinical variables. Multiple responses are generated by inflammatory cascades mediated by various molecules such as cytokines, adhesion molecules, chemokines, and cells such as lymphocytes T and B lymphocytes, leading to a final common pathway that predominantly affects the joints and structures of the synovial membrane, articular cartilage, and bone [2, 3]. The factors associated with the development of the disease comprise genetic and environmental factors. These include female gender, family history of RA, age, smoking, and genetic factors, including the Human Leukocyte Antigen (HLA) and non-HLA such as polymorphisms of PTPN22, STAT-4, tumor necrosis factor-*α* (TNF-*α*), T-cell receptor (TCR), and Fc-*γ* receptor (Fc-*γ*R), among others. The evidence on genetic susceptibility pointed at RA is mainly obtained from cohorts of Caucasians and Asian patients. Among white individuals who have RA, 80% express HLA-DR1 or HLA-DR4 [4]. However, there are few available data about the role of these genetic polymorphisms in the prevalence or severity of RA in the Afrodescendants (AD), who have evolutionarily and by natural selection developed mutations that allowed them to acquire resistance to infectious diseases like malaria. Some of the mechanisms by which this resistance was developed as a product of natural selection are involved in different forms of immunological pathways, many of them of a well-known importance in the pathophysiology of RA. This paper focuses on presenting the known mechanisms of resistance to malaria and their possible contribution to the pathophysiology of RA in this special group of patients.

## 2. The Role of Evolution and Natural Selection in the Development of the Immune System and Autoimmunity Phenomena

The human beings as well as all living species undergo evolutionary changes in response to natural selection in order to become more competitive favoring their survival. The immune system evolved primarily for defense against microorganisms and is comprised of various mechanisms that have been added over time. One of the older mechanisms is phagocytosis, characteristic of unicellular amebiform organisms. These ancient forms and primitive unicellular organisms acquired the ability to adhere and generate forms of intracellular signaling and specialization, thus generating cells with various functions configuring multicellular organisms or metazoans [5]. Some cells continued to perform the function of phagocytic cells (sentinel) that we recognize today even in complex animals, such as mammals. Then through this tendency to cellular specialization, T cells and various molecules were generated to fulfill the work of defense such as cytokines or complement proteins. Through the time, mechanisms were added and a form of innate evolutionary memory was generated that together it has been called innate immune system. Later in the evolutionary process, it also acquired the ability to generate an immunological memory for the life of the individual, to recognize and eliminate pathogens in the future for those which are not innate forms of elimination. Thus either the acquired or adaptive immune system appears. The developing central nervous system which leads to the generation of other forms of memory is integrated in this process, which involves neurons and glial cells, implicated in tertiary forms of evolutionary memory, which in the context of immunity plays a very important role giving its connections to the immune and endocrine systems (neuro-endocrine-immune connections), that affects multiple forms of regulation and adaptation as well as various pathological conditions if there are changes in its performance. Individuals acquire social status and with it the development of a quaternary evolutionary memory or social memory, whose dysfunction causes various forms of diseases, including those related to the immune system [6]. In this form of social memory, cultural aspects of humans are involved with multiple biological consequences, such as those associated with migration and displacement. This affects the evolutionary biology of individuals. In this paper we must take into account the genotypic and phenotypic differences that affect the presentation of RA in AD that developed in different geographical areas, such as North and South America [7, 8]. 

All aspects of cumulative evolution in the immune system mentioned above were developed in parallel with mechanisms that generated their regulation. Phagocytosis does its job and deregulation of its homeostatic mechanisms can lead to the development of several diseases (e.g., hemophagocytic syndrome). Also, disorders arising from deregulation of the complement system cause tissue injury due to immune complex deposition (e.g., lupus erythematosus, etc.). The simplest explanation for the existence of these regulatory mechanisms is the evolutionary accumulation that occurs parallel to immune defense mechanisms, in order to avoid self-harm [9].

Some of the known mechanisms by which autoimmune diseases develop is the alteration in the function of regulatory mechanisms. The RA does not escape this type of pathophysiological mechanisms, showing changes in molecular mechanisms such as cytokines, chemokines and adhesion molecules and cellular alterations of T and B lymphocytes.

## 3. Mechanisms of Resistance to Malaria

The mechanisms that confer resistance to malaria are the result of evolutionary changes conditioned by natural selection, being these changes, the strongest form of evolutionary pressure known in the recent history of humans [10]. Most of these genetic changes confer resistance to malaria through mutations such as “loss of function” such as several hemoglobinopathies, which are the consequence of a punctual change of a nitrogenous base in one of the genes of hemoglobin. The major known changes that determine resistance or decrease in the severity of malaria caused by *Plasmodium falciparum *are the sickle cell trait (heterozygosity in the change of valine for glutamic acid at position 6 of the beta fraction of hemoglobin-Hb S) and beta-thalassemia (deficit in the synthesis of the beta chain of hemoglobin) [11]. These changes create inhospitable conditions to the parasite that needs in red blood cell, the appropriate concentrations to survive, which are provided by normal hemoglobin. Other genetic alterations such as enzymopathies also confer resistance to malaria (deficiency of glucose-6-phosphate dehydrogenase or pyruvate kinase) [12].

Interestingly the number of modified evolutionarily molecules play important roles in the immune system. For example, a parasitic infection, as complex as Plasmodium, involves many immunological factors for its control. It depends on epidemiological factors, genetics, age, stage and parasite species, and duration of infection, among others. An individual can have multiple resistance factors and their combination determines different clinical presentations of infection. The immune response to acute infection, chronic exposure, or reinfection depends on changes that have occurred in the mechanisms of acquired immunity given by T and B lymphocytes. Specific antibody production can reduce for example, the severity of symptoms and mortality [13]. 

Several polymorphisms involved in immune response have been associated with resistance to malaria. Polymorphisms of class I HLA (HLA-Bw53) and class II HLA haplotype DRB1*1302-DQ B1*0501 are involved in resistance to malaria caused by *Plasmodium falciparum* [14]. The polymorphism T232 of the Fc gamma receptor IIB (Fc*γ*RIIBT232) also confers resistance to malaria, particularly to severe forms of infection (cerebral malaria) caused by *Plasmodium falciparum* [15]. 

The complement receptor 1 (CR1, also called CD35) is a protein of 190–280 kDa located on the surface of red blood cells and belongs to the complement regulatory protein, responsible for removing immune complexes from the circulation [16]. Some studies have suggested that PfEMP (*P. falciparum* erythrocyte membrane protein-1P), a membrane protein of *Plasmodium falciparum,* is a protein ligand of the CR1 and it could be involved in parasite rosette formation around erythrocytes, observed *in vitro* in forms of severe malaria and it could be also a pathogenic factor in these particular forms of disease [17]. The CR1 gene encoding presents several polymorphisms that are involved in the amount of protein expression in the red cell membrane, and it would presumably confer protection, but reports in the literature are contradictory [18, 19], conferring protection apparently to severe forms of the disease such as the cerebral malaria.

The TNF-*α* is a cytokine with proinflammatory activity. It is produced mainly by monocytes and macrophages although T and B lymphocytes may also produce this cytokine. It plays an important role in inflammation and is relevant to autoimmune and infectious diseases [20]. Several studies have suggested that TNF-*α* is an important mediator in the complications that accompany severe malaria by *P. falciparum* [21]. Two gene promoter polymorphisms of TNF-*α* (homozygosity for the TNF-308A [22] and alleles of TNF-376A [23]) have been associated with cerebral malaria in children in Gambia and Kenya (Africa).

In the case of infections caused by *Plasmodium vivax* the most studied evolutionary change that determines resistance is the genetic polymorphism of parasite receptor known as Duffy Antigen Receptor for Chemokines (DARC) [24, 25], in which form of absent alleles does not allow the receipt and entry of the parasite to the red cell.

## 4. Mechanisms of Resistance to Malaria and Their Possible Role in the Presence and/or Severity of RA

Based on case reports and series of patients with sickle traits and beta-thalassemias, there are no changes in the presence or in the natural history of RA. Clinical presentation of RA may occur slightly more severe for their possible association with arthropathy associated with sickle cell disease [26]. Little information is available about the role of asplenia (a condition related to these hemoglobinopathies) and RA. In the spleen, the immune complexes are cleared and the asplenia induced by hemoglobinopathies may influence the clinical course of diseases where these complexes play a major pathogenic role [27]. To our knowledge there are not reports in the medical literature of patients with enzymopathies as deficiencies of glucose-6-phosphate dehydrogenase or pyruvate kinase, and the relation to the development or progression of autoimmune diseases, except as related to the worsening of anemic condition common in these pathologies [28].

In the same perspective, there is no information that associates either the HLA class I, HLA-Bw53 or HLA class II haplotype DRB1*1302-DQ B1*0501, with RA or other autoimmune or inflammatory phenomenon such as spondyloarthropathies [29].

There are three classes of Fc*γ*R: Fc*γ*RI (CD64), Fc*γ*RII (CD32) and Fc*γ*RIII (CD16). Fc*γ*RII class has several isoforms named as IIA, IIB, and IIC. All these receptors except Fc*γ*RIIB isoform have activating functions given the presence of “ITAM” (immunoreceptor tyrosine activation motif) able to phosphorylate through the help of protein-tyrosine kinases (PTK), and thus they lead to the activation of intracellular proteins that are designed to promote the action of transcription factors that induce the formation of proinflammatory cytokines and amplification of the immune response [30].

Fc*γ*RIIB isoform has inhibitory function by possessing “ITIMs,” which are inhibitory or regulatory forms of the immune response [13]. The substitution of threonine for isoleucine at position 232 in the transmembrane domain known as Fc*γ*RIIBT232 determines an alteration in the function of this regulator receptor, which leads to an increase in immune reactivity mainly mediated by B lymphocytes and it is associated with the development of autoimmunity in experimental models and in humans. This polymorphism has also been correlated with the development of less severe malaria in animals and humans [15, 27]. Evolutionarily, a resistance to severe malaria has occurred in this case, but an increase in the development of autoimmune phenomena such as systemic lupus erythematosus (SLE) has been observed [31–35]. It is unknown whether this polymorphism is related to the development of RA, where the role of B lymphocytes is crucial for their development [36].

CR1 deficiency has been also associated with susceptibility to SLE [37], whose pathogenic factor would be the lack of clearance of immune complexes. A special relationship in RA patients has not been found yet. Some patients with RA show a decrease in expression of CR1 on the red blood cells, but it seems to be an acquired phenomenon [38]. 

Polymorphisms of TNF-*α* have been associated with the development of RA [39]. TNF is a fundamental cytokine in the development of RA. Furthermore, TNF-308A single nucleotide polymorphism has been associated with radiological damage in RA patients. This association may be due to higher production of TNF associated with this polymorphism. Pharmacogenomics may be important because some of the TNF polymorphisms are associated with low response to anti-TNF therapies [40]. 

Concerning the susceptibility to *Plasmodium vivax* infection, there are two kinds of alleles related to the receptors DARC: Fya and Fyb, which identifies four possible phenotypic presentations: homozygous Fy (ab−) (absence of the receiver or null), homozygous Fy (a+ b+) and heterozygous Fy (a− b+) and Fy (a+ b−). These receptors belong to the family of seven transmembrane molecules, initially recognized as a receptor for the *Plasmodium vivax* in human red blood cells and the simian *Plasmodium Knowlesi* and then recognized as a “promiscuous” receptor which is able to bind both CC and CXC chemokines and to have a cleaner role of these molecules [41]. Several chemokines related to the inflammatory process of RA are ligands of DARC, such as the CXC type chemokine, the interleukin-8 (IL-8) and neutrophil-activating protein derived from epithelial cells (ENA-78: epithelial cell-derived neutrophil-activating protein-78), and the CC type, such as chemoattractant protein of monocyte (MCP-1 monocyte chemoattractant protein-1) and the regulated protein of T lymphocytes expressed and secreted in normal activation (RANTES: regulated on activation normal T-cell expressed and secreted). The DARC expressed on endothelial cells of the synovium is important for the recruitment of neutrophils in patients with RA [42–50]. However, the role of DARC in the pathogenesis of RA is unknown. As it had been already mentioned, the DARC in this location has a role in clearance of circulating chemokines, a condition that could have an implication in the regulation of inflammatory processes. The phenotypes of the DARC have been studied in Caucasian patients from Italy with Behçet's disease, where the chemokines IL-8 and MCP-1 may be important in the pathogenesis. In this study, there was no association among the genetic polymorphisms of these genes and the presence of the disease [51]. It can be assumed that the absence or deficiency of the DARC would be related to more severe forms of RA.

Finally, some subsets of intermediate affinity T cells have been identified as associated with resistance to malaria and also to an autoimmune-like condition induced during infection [52]. Although it is unknown whether these changes in cellular immunity are associated with RA, they can play, eventually, a role in individuals with this type of resistance to malaria.

Table 1 summarizes the mechanisms associated to malaria resistance, its role in regulating the immune system, and its possible role in autoimmune phenomena such as RA. 

## 5. Conclusion

The evolutionary pressure in African ancestrally exposed to malaria has conditioned several changes at the molecular and cellular level generating difficulties for the reception of the parasite, metabolism, its possible removal by immune mechanisms and regulation of the inflammatory response to avoid damage to vital tissues like the brain. Some of these known and other putative changes can modify the development and progression of autoimmune diseases such as RA. Many pathophysiological factors are common among individuals with RA; however, these factors may be particular in individuals with ancestral exposure to malaria and mechanisms of resistance to it.

## Figures and Tables

**Table 1:** Summary of the mechanisms of protection to malaria, and their possible association with rheumatoid arthritis pathogenesis.

Factor	Evolutionary modification	Resistance mechanism to malaria	Modified immunological pathway	Possible role in rheumatoid arthritis pathogenesis
Duffy antigen	Duffy-negative allele	Lack of expression of chemokines receptor	Chemokines sink	Amplification of immune response and lack of chemokines depuration

*β*-globin	Sickle hemoglobin	Suppression of parasite growth in red cells and enhanced splenic clearance of parasitized erythrocytes	Reduced parasite cytoadherence	Unknown. Increased expression of VCAM-1, E-selectin, and ICAM-1? Decrease of immune complexes clearance by asplenia.

Fc*γ*IIB	Substitution of threonine for isoleucine at position 232 in the transmembrane domain of Fc*γ*RIIB (T232)	Increased clearance of malarial parasites	Phagocytosis of plasmodium falciparum-infected erythrocytes. Differentiation of B lymphocytes	The abnormal function leads to an increase in immune reactivity mainly mediated by B lymphocytes

CR1	Polymorphisms of CR1 are involved in the amount of protein expression in the red cell membrane.	Reduced ability of *P. falciparum*-infected CR1-deficient red blood cells to form rosettes	Reduced ability of *P. falciparum*-infected CR1-deficient red blood cells to form rosettes, and less severe disease	CR1 is a complement regulatory protein, responsible for removing immune complexes from the circulation. Decreased of immune complexes clearance

NK1.1(−) andNK1.1(+) subsetsofTCR (int) cells	?	?	?	Autoantibody production?

GYPC	GYPC-deficient erythrocytes	Protection against EBA-140-mediated invasion by *P. falciparum* parasites	Binding receptor-parasite protein	?

CR1: complement receptor 1; GYPC: glycophorin C.
